# Selecting instruments for Mendelian randomization in the wake of genome-wide association studies

**DOI:** 10.1093/ije/dyw088

**Published:** 2016-06-24

**Authors:** Daniel I Swerdlow, Karoline B Kuchenbaecker, Sonia Shah, Reecha Sofat, Michael V Holmes, Jon White, Jennifer S Mindell, Mika Kivimaki, Eric J Brunner, John C Whittaker, Juan P Casas, Aroon D Hingorani

**Affiliations:** 1 Institute of Cardiovascular Science, University College London, London, UK; 2 Department of Medicine, Imperial College London, London, UK; 3 Centre for Cancer Genetic Epidemiology, University of Cambridge, Cambridge, UK; 4 Centre for Clinical Pharmacology and Therapeutics, University College London, London, UK; 5 Clinical Trial Service Unit and Epidemiological Studies Unit (CTSU), Nuffield Department of Population Health, Oxford, UK; 6 Research Department of Epidemiology & Public Health, University College London, London, UK; 7 Department of Non-communicable Disease Epidemiology, London School of Hygiene and Tropical Medicine, London, UK; 8 Genetics Division, Research and Development, GlaxoSmithKline, NFSP, Harlow, UK

**Keywords:** Mendelian randomization, genome-wide association study, biomarkers, causal inference

## Abstract

Mendelian randomization (MR) studies typically assess the pathogenic relevance of environmental exposures or disease biomarkers, using genetic variants that instrument these exposures. The approach is gaining popularity—our systematic review reveals a greater than 10-fold increase in MR studies published between 2004 and 2015. When the MR paradigm was first proposed, few biomarker- or exposure-related genetic variants were known, most having been identified by candidate gene studies. However, genome-wide association studies (GWAS) are now providing a rich source of potential instruments for MR analysis. Many early reviews covering the concept, applications and analytical aspects of the MR technique preceded the surge in GWAS, and thus the question of how best to select instruments for MR studies from the now extensive pool of available variants has received insufficient attention. Here we focus on the most common category of MR studies—those concerning disease biomarkers. We consider how the selection of instruments for MR analysis from GWAS requires consideration of: the assumptions underlying the MR approach; the biology of the biomarker; the genome-wide distribution, frequency and effect size of biomarker-associated variants (the genetic architecture); and the specificity of the genetic associations. Based on this, we develop guidance that may help investigators to plan and readers interpret MR studies.

Key Messages

- MR offers novel opportunities for reliable causal inference within the framework of observational research designs.
- The findings from an MR analysis can provide insight into the pathophysiology of complex disease and have translational relevance, including the prioritization of drug targets.
- The emerging genetic architecture of disease biomarkers now allows more informed selection of genetic variants for MR studies than was hitherto possible.
- As the number of biomarker-associated variants grows though genome-wide association studies and, more recently, metabolomics and proteomics, selection of the most appropriate instruments for MR analysis will become an increasingly important issue.
- We have proposed a set of principles that should inform the selection process to aid the design, analysis and interpretation of MR studies.

## Introduction

Adverse environmental influences, such as smoking and alcohol consumption, are associated with a higher risk of many chronic, non-communicable diseases. Individuals at higher risk also exhibit alterations in numerous quantitative biological traits (also known as disease biomarkers or intermediate phenotypes), years before disease onset (Supplementary Table 1, available as Supplementary data at *IJE* online). These associations have been identified mainly through non-genetic observational studies. However, observational epidemiological studies of this type can be subject to a variety of biases. Importantly, it can be difficult to separate causal associations from those that arise from confounding or reverse causation. Effect estimates from such studies may also be prone to regression dilution bias^1^ and errors in the measurement of the biomarker for technical or biological reasons.^1^

**Table 1.** Illustrative examples of different types of MR study. Examples are provided of MR studies of exogenous exposures, *cis*-MR for drug target validation, and disease biomarker MR analysis

Author (year)	Location	Date of relevant GWAS	Exposure	Endpoint	Sample characteristics	Source of variant(s)	No. of variants	Genes	Hypothesized effect shown?	Formal MR methods	Meta-analysis	Total *n* (cases/controls)
Bech (2006)^117^	Denmark	2011	Caffeine intake	Stillbirth	Pregnant women	Candidate gene	3	*NAT2, CYP1A2, GSTA1*	Yes	No	No	299 (142/157)
Holmes (2014)^76^	UK	2011	Alcohol intake	CHD	General population	Candidate gene	1	*ADH1B*	Yes	Yes	Yes	261991 (20259/168731)
Sofat (2010)^118^	UK	–	CETP inhibition	Blood pressure	General population	pQTG	2	*CETP*	No	Yes	Yes	58948
Swerdlow (2014)^91^	UK	–	HMG-CoA reductase inhibition	Type 2 diabetes	General population	Candidate gene	2	*HMGCR*	Yes	No	Yes	223,463 (26236/164842)
Swerdlow (2012)^90^	UK	–	Interleukin-6 signalling	CHD	General population	pQTG	3	*IL6R*	Yes	No	Yes	133449 (25458/100740)
Rasmusen-Torvik (2010)^119^	USA	2008	Fasting glucose	Carotid IMT	General population	GWAS	5	*GCKR, G6PC2, GCK, SLC30A8, MTNR1B*	Yes	Yes	No	7260
Elliott (2009)^82^	UK	2008	CRP	CHD	General population/ case-control	GWAS	1	*IL6R*	Yes	Yes	Yes	46434 (14365/32069)
Giltay (2009)^120^	Netherlands	2008	Cholesterol	Depressive symptoms	Elderly men	Candidate gene	1	*APOE*	No	No	No	1089
Lim (2009)^121^	Singapore	2006	Obesity	Cataract	General population	Candidate gene	1	*FTO*	No	No	No	3000 (1339/1661)
Linsel-Nitschke (2008)^122^	Germany	2008	LDL-C	CHD	General population	Candidate gene	1	*LDLR*	Yes	Yes	No	7579 (1324/6255)
Perry (2009)^123^	UK	–	Beta-carotene	Diabetes mellitus	Case-control	Candidate gene	1	*BCMO1*	No	Yes	No	10128 (4549/5579)
Trompet (2009)^124^	Netherlands	2008	Cholesterol	Cancer	Elderly population	Candidate gene	1	*APOE*	No	No	No	2913 (290/2623)

pQTG, protein quantitative trait gene; LDL-C, Low density lipoprotein cholesterol; IMT, intima-media thickness.

**Figure 1.**
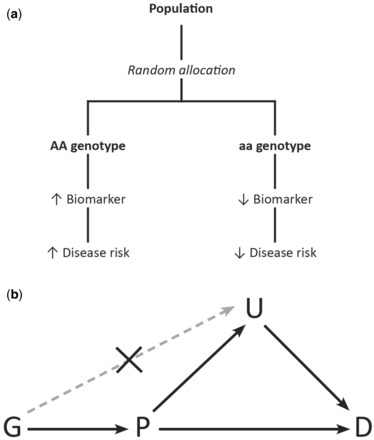
A: Mendelian randomization is a natural analogue of the classical randomized controlled trial (RCT). Random allocation of alleles at conception and the unidirectional flow of information from DNA sequence to endogenous biomarker phenotype allow causal inference of the type possible within the RCT framework. Genotype is generally unrelated to environmental exposures, thus reducing confounding. B: the Mendelian randomization model: the causal role of an exposure, P, on a disease state, D, is being evaluated. A genetic variant, G, is associated with biomarker P but not with confounders, U. Variant G is also associated with disease D and acts only through its effects on biomarker P. The model rests on three core assumptions: (i) the genetic instrument (G) is associated with the exposure or biomarker of interest (P); (ii) the genetic instrument (G) is independent of potential confounding factors (U) in the relationship between the exposure/biomarker (P) and the outcome (D); (iii) the outcome (D) is associated with the genetic instrument (G) only through the effect of the exposure/biomarker (P), and is in all other respects independent.

Mendelian randomization (MR) is an evolving paradigm in which genetic variants (usually single nucleotide polymorphisms, SNPs) are used to help distinguish causal from non-causal associations between environmental exposures or biomarkers and disease outcomes.^2^ Two unique attributes of genotype make this possible. First, the random allocation of parental alleles to zygotes at meiosis, independent of environmental exposures, reduces the potential for confounding in genetic association studies in the same way as randomized treatment allocation in clinical trials^3^^,^^4^ (Figure 1a). Second, the invariant nature of the DNA sequence and unidirectional flow of biological information, from gene sequence through intermediate phenotypes to disease, avoids reverse causation, though it should not be taken to imply a stability of genetic effect which in theory could be modified in a context-dependent fashion.^5^

An MR study typically considers three types of association: (i) the association of a biomarker (or environmental exposure) with the disease outcome; (ii) the association of a genetic variant with biomarker or environmental exposure; and (iii) the association of the same variant with disease risk^6^ (Figure 1b). Provided certain assumptions are met (Figure 1), consistency in direction and magnitude of the three estimates provides evidence on causal relevance of the environmental exposure or biomarker. The causal effect can be quantified within a formal statistical framework, using instrumental variables methods which have been adopted and adapted from the econometric literature.^7^^,^^8^ Some illustrative examples of the early use of MR are outlined in Box 1 and Table 1, and more recent examples that have exploited certain enhancements to the MR approach, are described in more detail later in this. It is notable that several important MR studies of certain disease biomarkers have identified inconsistency between effect estimates obtained in non-genetic observational studies and those through MR analysis that have altered thinking on the causal relevance of those biomarkers, as we describe later.

BOX 1. Applications of Mendelian randomization

MR analysis has been applied to assess whether CRP, a circulating marker of inflammation, plays a true causal role in the development of CHD. Despite the robust association of CRP level with CHD in observational studies, *CRP* variants used to instrument long-term elevations in CRP concentration did not provide evidence of a causal role for this biomarker in the development of CHD, based on meta-analysis of up to 47 studies including 46 557 cases.^78^^,^^82^^,^^125^ The observational association between CRP and CHD is more likely explained by confounding or reverse causation. HDL-cholesterol (HDL-C) exhibits an inverse association with CHD risk in observational studies, but whether this association is causal has been in dispute. An MR study used variants in the *LIPG* gene, encoding hepatic lipase, as an instrument for HDL-C and examined its relationship with myocardial infarction (MI) risk.^104^ Although higher HDL-C is observationally associated with lower MI risk, MR analysis based on *LIPG* variants, both alone and within allele scores to instrument HDL-C concentration, did not find evidence for a causal role for HDL-cholesterol in CHD.

A systematic review (see Supplementary methods for details, available as Supplementary data at *IJE* online) reveals a 10-fold increase in MR studies published between 2004 and 2015 (Supplementary Figure 1, available as Supplementary data at IJE online). The majority have been in the fields of cardiovascular disease and diabetes (51% of published studies); other disease areas including cancer (10%); and mental health (10%). Most MR studies (86%) have been of disease biomarkers (defined in Box 2) such as blood lipids, body mass index (BMI) or blood pressure, and 50% have used a candidate gene approach to identify suitable instruments (Table 1). However, genome-wide association studies (GWAS) of disease biomarkers are providing a new source of instruments for MR analysis. Of the 2111 GWAS listed in the NIH National Human Genome Research Institute (NHGRI) GWAS catalogue^9^^,^^10^ [http://www.genome.gov/gwastudies, as of 23 August 2015], 672 (32%) concern genetic variants associated with 520 disease biomarkers, with some variants exhibiting associations with more than one biomarker. Other studies based on high-density locus-centric SNP arrays such as Metabochip^11^ and Immunochip^12^, designed based on GWAS findings in cardiometabolic and autoimmune/inflammatory disorders respectively, have reported many additional genotype-biomarker associations. Many MR studies (*n* = 211) were published after a GWAS of their corresponding biomarker; of those studies, 61% (*n* = 129) used the preceding GWAS to inform the selection of the instruments.

BOX 2. A hierarchy of biomarkers for Mendelian randomization studies based on the central dogma

**Figure 2.**
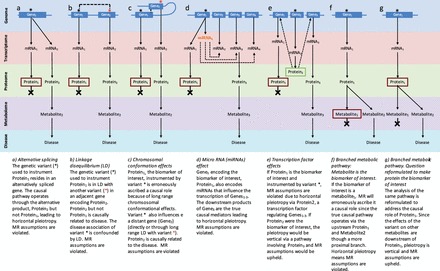
Mechanisms that may give rise to genetic pleiotropy and implications for MR analysis.

For the purposes of this review, we separate exposures that might alter disease risk that are external (exogenous) to the body (e.g. cigarette smoke or socioeconomic position) from those that are internal to the body (endogenous), which we refer to as disease biomarkers. We recognize a hierarchy of disease biomarkers that reflects the central dogma—the unidirectional information flow from gene through mRNA to protein. The influence of genetic variation is initially on mRNA sequence or level, and then on the function or amount of the encoded protein. Such alterations in proteins then lead to the downstream biochemical or structural alterations, including changes in more complex phenotypes (e.g. blood pressure) that affect disease risk. Among these endogenous exposures, we draw a natural distinction between proteins and more downstream biomarkers because proteins usually represent products of individual genes and are the most proximal, widely measured consequence of natural genetic variation (Figure 2).

Many of the early reviews in the field that covered the concept, applications^1^^,^^2^^,^^7^^,^^13–15^ and analytical aspects of the MR technique^16^^–^^18^ preceded the surge of GWAS. Thus, the question of how best to select instruments for MR studies, given the now extensive pool of available variants, has received insufficient attention. In this article we focus on the most common category of MR studies—those concerning disease biomarkers (see Box 2). We show that using GWAS as a source of instruments for MR analysis requires consideration of the assumptions underlying the MR approach, the biological nature of the biomarker of interest, the distribution of SNP-biomarker associations at the genome-wide and regional levels, the genetic effect sizes and specificity of associations.

## Assumptions underlying MR analysis

The MR approach, as classically described, rests on the assumption that any disease association of a genetic variant employed as an instrument because it proxies the biomarker of interest should be both unconfounded and explained exclusively through an effect on the biomarker (Figure 1b).^15^ A potential violation of these assumptions occurs when an SNP associates with several biomarkers, only one of which is of causal interest. The association of a genetic variant with more than one phenotype is commonly referred to as pleiotropy. When pleiotropy is observed, two of the three critical assumptions of an MR analysis may be called into question. However, as we show later, a pleiotropic variant need not necessarily be excluded as an instrument, provided careful consideration is given to the mechanism giving rise to the pleiotropy and to the nature of the biomarker of interest; specifically, whether or not this is a protein. We also evaluate a number of enhancements to the basic MR design, based on multiple instruments which have since been developed partly to enhance power of MR studies, and partly to overcome some of the challenges imposed by pleiotropic instruments.

## Disease biomarkers and their position in the putative disease pathway

Interest in some disease biomarkers is in their performance as predictors of disease risk.^1^ For this application, it is not essential that the biomarker-disease association is causal; merely that there is a demonstrable and consistent association of the biomarker with the disease, that is of sufficient magnitude to make it a useful predictor. However, if there is interest in the potential aetiological role of a biomarker that might be amenable to modification by public health measures or drug treatment, evidence on a causal association is essential. Thus, reliable demonstration of even a modest causal effect through genetic association analysis could still be important because of the potential to develop interventions with a much larger effect on the same biomarker.^2^^,^^16^

Disease biomarkers are biologically diverse, encompassing circulating proteins (e.g. fibrinogen, C-reactive protein or interleukin-6), low molecular weight metabolic intermediates (e.g. homocysteine and uric acid) and complex physiological phenotypes such as blood pressure (Supplementary Table 1). Most biomarkers are continuous traits with genetic and environmental determinants. Many follow an approximately normal (or log-normal) distribution, and show a linear (or log-linear) association with disease risk. As we show later in a detailed discussion of potential reasons for genetic pleiotropy, the position of the biomarker of interest in the pathway connecting genetic variation to disease risk has an important bearing on the design, interpretation and validity of an MR study. In particular, we show why MR analysis of protein biomarkers instrumented by SNPs in the encoding gene has certain advantages over other categories of MR analysis.

## Genetic architecture of SNP-biomarker associations

The wealth of GWAS findings allows some observations to be made about the genetic architecture of different disease biomarkers, which has bearing on the selection of SNPs for MR analysis of these traits. However, it must be borne in mind that most previous GWAS have utilized genotyping arrays that have a bias towards common variants, so that there is less information on alleles of lower frequency and their potential role as instruments in MR analysis.

C-reactive protein (CRP), an acute-phase protein associated in observational studies with cardiovascular disease (CVD) risk, provides an illustrative example (Box 1). Three ‘Manhattan’ plots (Supplementary Figure 2, available as Supplementary data at *IJE* online) depict genetic associations with CRP: the first is based on findings in 5000 participants from the Whitehall II study,^19^ genotyped using a gene-centric 50000 -SNP array (IBC HumanCVD BeadChip ‘Cardiochip’) covering 2100 genes implicated in CVD;^20^ the second is from a GWAS in 6345 participants from the Women’s Genome Health Study;^21^ and the third is from a subsequent meta-analysis of GWAS of CRP including 82 725 participants from 15 studies.^22^ The findings illustrate some general features of genomic associations with biomarkers.

First, genetic associations with mRNA expression or protein biomarkers such as CRP may be detected with smaller sample sizes when compared with studies of disease endpoints, presumably because the level or function of a protein biomarker is a comparatively proximal consequence of genetic variation, with fewer biological steps between DNA sequence variation and protein synthesis and a larger signal-to-noise ratio.^23^ For more distal biomarkers such as blood metabolites or complex physiological phenotypes such as blood pressure, larger samples have typically been required. Nevertheless, regardless of the type of biomarker, increasing sample size, usually through meta-analysis, leads to identification of additional associated variants. Low-frequency variants, such as those identified by newer exome and whole genome sequencing studies sometimes of larger effect than common variants studied in GWAS, but common alleles can also on occasion produce large effect sizes. However, whole genome arrays are mainly populated by common alleles and even imputation against the 1000 Genomes reference panel most efficiently captures information on other common rather than rare alleles. Therefore, the new loci detected later in larger GWAS datasets tend to also harbour common variants but with smaller effects than the loci identified by earlier, smaller studies. For example, when 25 independent GWAS of CRP were pooled by meta-analysis, with an aggregate sample of 82 725 individuals, 12 additional loci were identified beyond the 7 reported by an earlier, smaller study. The effect sizes at each of these new loci were generally smaller than in the sentinel study (Supplementary Figure 3, available as Supplementary data at *IJE* online).^22^ Meta-analyses of GWAS of blood lipids,^24^ BMI,^25^ blood pressure^26^ and other disease biomarkers have also led to the identification of new loci also generally of smaller phenotypic effect, undetected by earlier, smaller GWAS.

**Figure 3.**
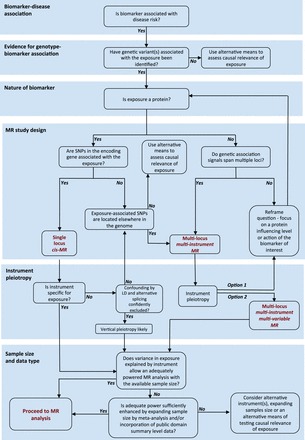
Illustrative guide to some of the key decisions in selecting instruments for MR analysis of disease biomarkers, based on the principles outlined in this review. The figure is intended to help plan a Mendelian randomization study of a disease-associated biomarker and should not be viewed as an inflexible decision tree. For additional considerations and details, please refer to the main text.

Second, loci containing genetic variants associated with CRP are scattered throughout the genome. This appears to be a general feature of loci associated with disease biomarkers, such as circulating metabolites (e.g. homocysteine^27^ and uric acid^28^^,^^29^), lipoproteins,^30^^–^^32^ metabolomic profiles^33^^–^^36^ and the more complex physiological phenotypes such as blood pressure^26^^,^^37^^,^^38^ and BMI.^25^^,^^39–46^ For protein biomarkers like CRP, a natural and important distinction emerges between two categories of genetic variants that might be used for MR analysis. The first are those variants acting in *cis*, located in the vicinity of the encoding gene (in this case *CRP*, chr1q23.2), which are potentially coincident with *cis*-eQTLs (expression quantitative trait loci) influencing mRNA expression. GWAS of mRNA expression profiles and protein biomarker concentrations indicate that *cis*-acting variants are a common feature of the genome.^47^ The second category contains those acting in *trans*, i.e. located outside the gene encoding the protein biomarker of interest, often on a different chromosome. A variant at one locus associated with an effect on expression of a distant gene may operate via chromosomal conformational mechanisms, through microRNAs that alter mRNA stability of a range of distant target genes,^48^ or because they are located in genes encoding transcription factors that regulate expression of other physically distant genes^49^ or by downstream biochemical mechanisms. It is SNPs of this type that can often be pleiotropic.

Third, it is typical not only to identify associations with biomarkers at widely separated genomic locations, but also to observe multiple biomarker-associated SNPs at each locus. Although multiple independent causal variants may be present at a single locus, the multiplicity of associations commonly arises due to linkage disequilibrium (LD) between SNPs, only a subset or one of which may be functional. In order to use a SNP as an instrument in MR analyses, it is not necessary to prove the SNP itself is the causal variant, provided that its association with the biomarker of interest arises from LD with a causal variant within the same locus. Moreover, and importantly, there must be no additional LD with other nearby variants that might influence the expression or activity of a different protein. Were that the case, LD would lead to confounding and violate a key assumption of the MR paradigm. Both local LD (i.e. in the immediate vicinity of a given SNP) and distant LD (i.e. elsewhere on the same chromosome) can be ascertained using web-based tools such as the SNP Association and Proxy Search (SNAP) resource at [http://www.broadinstitute.org/mpg/snap/^50^]. In the CRP example, the *CRP* gene^51–55^ is isolated by two recombination hotspots (Supplementary Figure 4a, available as Supplementary data at *IJE* online) with no evidence for LD with SNPs in the adjacent *DUSP23* and *APCS* genes. This substantially reduces the risk that confounding by LD would compromise MR analysis using SNPs in the *CRP* gene. SNP selection for an MR analysis becomes more challenging where multiple SNPs are in LD, all associate with the biomarker of interest and the associations span several genes in close physical proximity. For example, a 74kb region of chromosome 1 (chr1p36.2) contains the *MTHFR*, *NPPA* and *NPPB* genes and includes SNPs associated with circulating concentrations of homocysteine, atrial- and brain--type natriuretic peptide, each of which have been implicated as causal factors in cardiovascular disease^56–58^ (Supplementary Figure 4b). Statistical methods for prioritizing SNPs in such circumstances, such as conditional analysis or variable selection, are available and have been described elsewhere.^59^^,^^60^ Recent developments include a Bayesian statistical test to quantify the probability that associations observed at the same locus with a range of outcomes (e.g. mRNA expression, blood a biomarker disease outcome) can be explained by the same causal variant,^61^ which may help map association signals from a GWAS to the responsible gene. However, functional annotations or experimental evidence may be required in some cases to support the selection of instruments.

## Genetic effect size

SNP arrays deployed in GWAS contain common variants (minor allele frequency, MAF, > 5%), which tend to have small to moderate effect sizes.^62^ Statistical analyses in GWAS set stringent significance thresholds (typically *P*-value < 5 x 10^-8^) in order to reduce the number of false-positive associations arising from the vast number of statistical tests performed. For this reason, and because false-positive associations were a feature of the era of candidate gene association studies,^63^ much attention in a GWAS is correctly on the reliability of any genetic association, based on the *P*-value.

Provided an association is identified robustly, the size of the genetic effect gains importance when prioritizing SNPs for use as MR instruments, with SNPs of larger effect preferred because they increase statistical power provided the minor allele frequency is sufficiently high.^7^ In a study with a fixed sample size, the *P*-value for the SNP-biomarker association provides an indirect measure of the effect size, but this is also influenced by the frequency at which the variant occurs in the sample, and the LD relationship between the typed variant and the causal variant (if they differ).

Specific metrics of effect size can be used to inform the selection of SNPs as instruments in an MR analysis. The most commonly used indicators of effect are: (i) the beta-coefficient from a linear regression of each additional minor allele of an SNP locus with the trait of interest, which equates to the absolute difference in concentration of biomarker for each additional allele, expressed on the native or standardized scale; (ii) the proportion of the phenotypic variance explained by the SNP in the sample (R^2^); and (iii) the F-statistic from the linear regression model of the genetic instrument with the biomarker. Both R^2^ and the F-statistic are influenced by the minor allele frequency, and the F-statistic is additionally affected by the sample size,^64^ For the F-statistic, an arbitrary threshold value of F > 10 has been proposed for determining suitability of SNPs for MR analysis,^7^ to avoid weak-instrument bias. However, investigators should be cautious about the use of an arbitrary F-statistic threshold for the selection of instruments, particularly where the estimate of the F-statistic comes from a single small study. As reported by Burgess and Thompson,^65^ F-statistic estimates can be inflated by chance in small studies. This is because ‘confounders may not be perfectly balanced between genotypic subgroups in finite samples’.^65^ Under such circumstances the chance difference in confounders may explain more of the difference in the biomarker of interest between the genotypic groups than the instrument itself. As a corollary, the estimate of the causal association will be inflated towards that of the biased observational association between the biomarker and disease outcome. Since the F-statistic is related to the proportion of the variance in the biomarker explained by the genetic variants, the sample size and the number of instruments, Burgess and Thompson suggest three ways in which this effect can be mitigated: by increasing the sample size and/or by combining genotype biomarker associations across studies by meta-analysis; by increasing the number of instruments; and by adjusting for measured covariates. Many of these approaches are now routinely applied in contemporary MR analysis

Returning to the CRP example, Supplementary Figure 5 (available as Supplementary data at *IJE* online) illustrates how the choice of effect metric affects the ranking of potential SNPs that might be used as instruments in an MR analysis. In general, low-frequency variants with large effects tend to rank highly when assessed using the beta-coefficient, but diminish in priority when ranked by the proportion of variance explained (R^2^) or F-statistic, because the latter penalize low allele frequency. In general, we have found the proportion of variance explained (R^2^) to be the most useful metric of effect when planning SNP selection for MR analysis. For these reasons, most successful MR analyses to date have relied mainly on common variants as instruments. Rare variants are of value in other types of study design, including recall-by-genotype studies.

Though common SNPs typically explain only a small proportion of the variance in a trait, the value of R^2^ should be placed in context. For example, a common SNP (rs1205) in the vicinity of the CRP gene (MAF = 0.34) explains only 0.7% of the variance in this trait, but the difference in CRP concentration per allele (beta-coefficient: -0.15mg/l log CRP) is similar in magnitude to the difference in CRP value between treatment and control groups in a randomized clinical trial of rosuvastatin, a potent statin drug which lowers CRP in addition to its effect on blood lipids.^66^

The degree to which loci contribute to biomarker variance may also vary. For some biomarkers, a single locus may dominate (e.g. *LPA* associated with lipoprotein(a) concentration).^67^^,^^68^ In other cases, gene-centric and genome-wide analyses of uric acid^29^ and HDL-cholesterol^31^^,^^32^^,^^69–71^ indicate that SNPs at multiple loci contribute to the variance in each trait but certain loci harbour variants of large effect [*SLC2A9* and *CETP*, respectively, (Supplementary Figure 6, available as Supplementary data at *IJE* online)]. In our experience, SNPs acting in *cis* with effects on mRNA transcription level and protein concentration are often, but not invariably, those with the largest effect^21^.

## Specificity of the genetic association

The assortment of alleles at the time of gamete formation is independent of environmental factors. This is why genetic variants associated with disease biomarkers generally exhibit no association with behavioural, dietary and lifestyle factors, even though the biomarkers they instrument frequently do.^72^ However, we note that certain genetic variants have been identified that influence habitual behaviours such alcohol and coffee consumption or smoking.^73–75^ In such cases, these associations arise not because of non-random assortment, but rather because there is a mechanistic explanation: the variants influence the expression or function of genes involved in the handling of, or response to, chemical constituents of these exposures leading to an alteration in smoking or drinking behaviour. Such variants have in fact served as useful instruments to evaluate the causal influence of such exposures on the risk of common disease^76^^,^^77^ (Table 1).

A previous MR analysis of CRP (Box 1) using *cis*-acting SNPs in the CRP gene as instruments, had a particularly straightforward interpretation because variants in the gene were associated exclusively with the encoded CRP protein but none of the very wide range of other biomarkers with which CRP itself is associated.^78^ Similarly, specific genotype-biomarker associations were reported in an MR analysis of fibrinogen levels using SNPs in the *FGB* gene related to fibrinogen levels.^79^ However, because of the complex biological inter-relationships between the widely measured circulating biomarkers,^80^ biomarker-associated SNPs rarely exhibit the degree of specificity that was fortuitously observed with SNPs in the *CRP* gene; it is more common to find that SNPs identified for an association with one biomarker are also associated with several others. Speculatively, this issue is likely to become more prominent as a wider range of biomarkers are more routinely measured using new proteomic and metabolomic technologies.

For example, variants in *LIPC* (rs4775041, chr15q21.3) are associated with both HDL-cholesterol and triglycerides,^81^ and variants at the *APOE* cluster (rs4420638, chr19q13.32) with LDL-cholesterol, HDL-cholesterol, triglycerides,^69^ CRP,^82^ Lp(a) levels^83^ and lipoprotein-associated phospholipase A2 activity.^84^ At first glance, this lack of specificity might be interpreted as irrevocably violating one of the principal assumptions of the MR paradigm. For example, an association of the rs662799 SNP in the *APOA5* gene (chr11q23.3) with triglyceride level and coronary heart disease (CHD) risk^85^ may be taken to indicate a causal role in CHD for triglycerides. However, since this SNP is also associated with HDL-cholesterol, it is uncertain whether the CHD association of this SNP reflects its effect on triglycerides, HDL-cholesterol or some other consequence of variation in this gene.

The mechanisms responsible for pleiotropy may be varied, but have been incompletely characterized. However, based on available understanding of genomic organization and gene regulation, established or theoretical reasons for an association of a SNP with several biomarkers include (Figure 2):

- an effect on expression of an alternatively spliced gene leading to two distinct protein products with different actions (Figure 2a);
- linkage disequilibrium between SNPs spanning nearby genes at the same locus (i.e. the problem of confounding by LD) (Figure 2b);
- an effect in *cis* on expression and in *trans* (either directly or through LD with an adjacent SNP) on a physically distant gene mediated through chromosomal conformational effects (Figure 2c);
- an effect on expression of a microRNA that regulates the stability of transcripts from multiple target genes (Figure 2d);
- an effect on expression of a transcription factor (e.g. hepatocyte nuclear factor-1α^86^) that regulates several distant target genes (Figure 2e);
- and residency of an SNP in a gene encoding a single protein whose activity influences several downstream biomarkers, some of which lie on the causal pathway to a disease outcome and some which may not (Figure 2f, g).

Two assumptions of an MR analysis are that the instrument should not be associated with any confounder of the biomarker disease association and that the association of the genetic instrument with the disease outcome should be mediated solely through the biomarker of interest. When a genetic variant is associated with several biomarkers, including the biomarker of interest, the assumptions of an MR analysis will be violated if the explanation for the pleiotropy is a disease pathway that branches more proximally to the biomarker of interest. This has been termed horizontal pleiotropy.^87^ By contrast, the assumptions of an MR analysis hold if the associations of a genetic variant with several biomarkers arise because of the serial and sequential effects of the biomarker of interest on others residing more distally on the same causal pathway to disease. This has been termed vertical pleiotropy. In Figure 2, we explain in more detail how the different established and putative mechanisms listed above could give rise either to horizontal or vertical pleiotropy, and the implications for MR analysis.

Thus, when faced with a candidate instrument that exhibits genetic pleiotropy, a critical issue for MR analysis is the likelihood that this is vertical rather than horizontal in nature. Confidence in a vertical explanation for pleiotropy may be high when there is good pre-existing functional insight. For example, the association of obesity-related gene variants with a range of cardiometabolic traits has been interpreted as evidence of the causal effect of adiposity on these other risk factors.^88^ In other cases, however, understanding of the functional relationships between the myriad circulating biomarkers may not be deep, and it may be difficult to exclude the possibility of horizontal pleiotropy. Moreover, the extent of horizontal pleiotropy may be underestimated because of the relatively modest number of biomarker measures that are currently available in epidemiological studies. The availability of new nuclear magnetic resonance^89^ and mass spectrometry-based lipidomic and metabolomics analysis will soon allow more comprehensive assessment of horizontal pleiotropy. However, such technologies also offer the enticing prospect of ascertaining genetic instruments that instrument certain circulating biomarkers more precisely. For example, the major blood lipid fraction HDL-C actually represents the cholesterol content of a wide range of high-density lipoprotein particles which each may have a different aetiological relationship with other lipids and metabolites and with disease risk.

The variety of mechanisms by which horizontal pleiotropy may arise are diminished the closer the biomarker of interest lies (in a functional sense) to the genetic variant which is acting as the instrument, hence the importance of considering the nature of the biomarker of interest in an MR analysis. According to the central dogma of molecular biology, there is a unidirectional flow of information from genetic sequence variation through mRNA, protein and thence through myriad downstream metabolic changes en route to disease events. In essence, invariant sequence variation in DNA can encode downstream perturbations in the transcriptome, proteome, metabolome and, in some instances, disease risk, whereas these perturbations cannot, to the best of our knowledge, alter DNA sequence. Sequence variation can therefore be envisaged as producing a series of sequential perturbations of the transcriptome, proteome and then metabolome. Proteins are the most widely measured proximal circulating biomarkers of interest for MR, separated from the genetic sequence only by mRNA. Thus, when a protein biomarker is instrumented in an MR analysis by *cis*-acting variants in the vicinity of the encoding gene, the likelihood of horizontal pleiotropy is diminished, though it is still possible (e.g. by alternative splicing of mRNA species; see Figure 2). If alternative splicing of the mRNA, the presence of a local miRNA encoding site and confounding by local and long-range LD can be reliably excluded (e.g. based on widely available, detailed, open access bioinformatic data), any pleiotropy observed of a *cis*-SNP instrumenting its encoded protein is more likely to be vertical than horizontal in origin. For this reason, MR analysis of protein biomarkers, based on *cis*-SNPs, forms a privileged category of MR analysis—which we term `*cis*-MR'. Proteins form the targets of most drugs, and several recent examples have demonstrated that variants in genes encoding a drug target mimic the mechanism-based consequences of modifying the same target pharmacologically,^90–92^ confirming the validity of the assumption of vertical pleiotropy and exemplifying the utility of *cis*-MR. This observation is motivating a particular use of *cis*-MR: for drug target selection and characterization, with applications in drug development.^93^ SNPs acting in *cis* could also be used as instruments to assess the causal relevance for disease of epigenetic marks such as DNA methylation^94^ or an even more proximal consequence of sequence variation, mRNA level.^23^

## Handling non-specific SNP associations in MR analysis of non-protein biomarkers

The lack of specificity of genetic associations poses greater difficulty when the biomarker of interest is not a protein but a more distal biomarker, for example a lipid particle (such as HDL-cholesterol) or a metabolite (e.g. uric acid). In such cases, the distinction of *cis*-SNPs from other categories of instrument is redundant. Moreover, because of limited functional understanding, it may be difficult to distinguish which of the several biomarkers associated with an SNP lies proximal to the biomarker of interest (and which could then influence disease independently of it, violating one of the MR assumptions), and which might lie distal to it on the causal pathway to disease (Figure 2). In effect, under such circumstances it can be difficult to distinguish horizontal from vertical pleiotropy. How can the problem of non-specificity of the available instruments be addressed in such situations? Three complementary approaches are considered, which harness the knowledge base of genome-wide associations with disease biomarkers or recent methodological developments.

## Demonstration of the consistency of SNP-biomarker-disease associations, regardless of the genetic instrument employed

The first option is to compare the effect on disease risk of genetic variants from different locations, each exhibiting a shared association with the biomarker of interest but with a different repertoire and pattern of effects on other biomarkers. Here, causality for the biomarker of interest would be inferred from a consistent association of the different instruments with both the biomarker and the disease outcome. For example, SNPs in *LDLR*, *PCSK9*, *APOE* and *SORT1*^31–33^^,69,70,95,96^ have a distinct repertoire of effects on other biomarkers but all associate with LDL-cholesterol and also with the risk of CHD events, in proportion to their effect of LDL-cholesterol, as carefully shown by Ference and colleagues.^97^ This consistency provides strong support for the causal role of LDL-cholesterol in the pathogenesis of CHD (Supplementary figure 7, available as Supplementary data at *IJE* online). By analogy, blood pressure was confirmed to be a causal factor in CHD because the many different blood pressure-lowering drugs tested in RCTs (including diuretics, beta-blockers and calcium channel blockers) each reduced CHD risk despite different mechanisms of action and different effects on other variables such as serum potassium, glucose and uric acid.

## Multi-locus approaches

A second approach, whose use has been growing,^88^^,^^98–100^ is to derive a new genetic instrument that incorporates information from multiple loci. The instrument is composed of SNPs selected from across the genome on the basis of a genome-wide significant association with a trait of interest, recognizing that some may exhibit associations with additional biomarkers. The most conservative approach is to select a single, strongly associated SNP from each locus; however, approaches that incorporate several SNPs at each associated locus where these are independent of one another, to a whole genome approach, including SNPs whose associations are below genome-wide levels of significance, have also been explored.^101^ The potential benefits are 2-fold. The first is an increase in the variance in the trait of interest explained by the genetic instrument to improve the power of the MR analysis. The second is a possible dilutional effect on pleiotropy, since SNPs selected on the basis of an association with one biomarker should not systematically be associated with other biomarkers unless one or more of these is in a related biological pathway. Under those circumstances, it would not be possible to eliminate pleiotropy entirely. The stability of the causal estimate based on a multi-locus gene score, to the exclusion of subsets of SNPs drawn at random, can be used as an adjunct means to evaluate bias in the causal estimate that may arise from the potential pleiotropic influence of a subset of SNPs.

As discussed previously, SNPs associated with a particular biomarker tend to be distributed across many independent, biologically distinct loci (e.g. at least 36 loci associate with LDL-cholesterol, 47 with HDL-cholesterol, 32 with triglycerides^30^ and 23 with blood pressure^26^^,^^37^^,^^38^^,^^102^). It is therefore possible to assign to each individual in a dataset a score based either on a simple count of the number of trait-raising alleles carried, or a score where the allele count is weighted by the per-allele biomarker effect size.^103^ The set of SNPs used for calculating such scores should have minimal redundancy so that each SNP is independent in its trait effect, a simple approach is to select a single SNP from each locus. In theory, associations that arise because of horizontal pleiotropy at one locus should then be independent of horizontal pleiotropic effects at other loci, and these smaller, unsystematic horizontal pleiotropic associations should be diluted relative to associations with the trait of interest. Supplementary Figure 8 (available as Supplementary data at *IJE* online) illustrates this effect using gene scores for HDL-cholesterol and triglycerides in a sample of 5000 men and women in the Whitehall II study.^19^ The scores were constructed using variants identified by one of the largest GWAS of lipids published to date^30^ and are robustly associated with their cognate lipid fractions. In each case, the score exhibits a considerably stronger association and greater specificity than any individual SNP (Supplementary Table 2, available as Supplementary data at *IJE* online), which has also been demonstrated in an analysis of allele scores for three clinically important biomarkers.^101^ A simple, unweighted score is justifiable if the component SNPs all exhibit similar effect sizes. However, where a small number of loci have a dominant effect on the trait of interest (as is the case with uric acid, for example), a weighted score may be preferable. For weighted scores, the effect size should ideally be calculated in a dataset independent from that used for the MR analysis, to reduce bias as a consequence of over-fitting.^17^

A further enhancement of the multi-locus approach has been to use information from multiple SNPs but to treat them as individual instrumental variables in a multivariable model, (see Palmer *et al.*^17^). Approaches that allow the incorporation of summary genetic effect estimates have also been developed, obviating the need to have access to participant-level data.^104^^,^^105^ Techniques have also been developed to accommodate the situation where genotype-biomarker associations are available in datasets distinct from, or only partially overlapping with, those in which genotype-disease associations are estimated.^106^

Despite the attraction of multi-locus approaches, it can still prove difficult to develop a truly specific genetic instrument. For example, a multi-locus MR analysis of the causal relevance of the three major lipid fractions was unable to identify instruments that were truly specific for each lipid fraction, the development of specific instruments for HDL-cholesterol and triglycerides being particularly problematic. Approaches developed to deal with residual pleiotropy include dropping the most pleiotropic SNPs from the instrument (with a corresponding reduction in power) or adjusting for residual pleiotropy in the analysis, which requires access to participant-level data and is unsatisfying conceptually as it returns to a standard observational approach that it was hoped would be rendered unnecessary by MR analysis.^100^^,^^107^^,^^108^

A further development, referred to as multivariable MR analysis, allows for vertical or horizontal pleiotropic associations among a pre-specified, measured set of risk factors.^109–111^ The assumptions of this approach are: that the genetic variants used as instruments are associated with at least one of a pre-specified set of risk factors, including the risk factor(s) of primary interest, but not with any others that might confound the association of the biomarker(s) of interest with the disease outcome; and that none has an effect on disease outcome except through the set of pre-specified risk factors. The approach has been applied to dissect the causal relevance of HDL-cholesterol and triglycerides for CHD, using summary effect estimates from previous GWAS.^107^ Although a clear advance, the approach can only allow for biomarkers that have been measured in the dataset. Horizontal pleiotropy due to unmeasured biomarkers may still undermine causal interpretation as with other types of MR analysis. The approach also focuses on the causal relevance of the biomarker of interest on the disease outcome independent of other biomarkers, which may underestimate the total causal effect in the preence of vertical pleiotropy operating through another biomarker in the pre-specified set.

To address the issue of unmeasured pleiotropy, Bowden *et al.*^112^ recently reported that Egger regression, originally developed to quantify small-study bias in meta-analysis of randomized trials, can be adapted and applied to provide an unbiased estimate of the causal effect of a biomarker on disease outcome even in the presence of invalid genetic instruments. Briefly, the unbiased causal effect of a biomarker on disease outcome is estimated as the slope of the regression line from a plot of the genotype-disease against genotype-biomarker association for a set of variants selected for an association with the biomarker of interest. By contrast to the more usual two-stage least squares regression, the Egger regression line is not constrained to pass through the origin. The intercept of the line provides an estimate of the extent of unmeasured pleiotropy. The approach is attractive but suffers from a reduction in power compared with the other methods. The reader is referred to the original paper for more details. Sensitivity analysis, in which effect estimates from standard two-stage least squares instrumental variable analysis, multivariable MR analysis and MR-Egger are compared, may help better judge the causal relevance of any given biomarker. This approach is illustrated in a recent MR analysis of uric acid in CHD.^113^

Importantly, regardless of the strengths and weaknesses of each of these approaches, and bearing in mind there may be no perfect solution to the problem of pleiotropic instruments in the MR analysis of non-protein biomarkers, all approaches can be considered to be a substantial advance over non-genetic observation studies.

## Reformulating the study question as a *cis*-MR analysis

A third approach to addressing pleiotropy is to reframe the research question so as to make a protein the primary `exposure' of interest. This allows the investigator to harness the advantages of *cis*-MR. Since *cis*-acting regulatory variants in the vicinity of genes that influence mRNA and protein expression appear to be a consistent feature of the genome, the genetic tools for *cis*-MR analyses of this type should generally be available. Moreover, since more than 90% of drug targets are proteins,^114^ the analysis is likely to have translational relevance, as *cis*-MR analysis has a role as a means for drug target validation. For example, a question on the causal role of HDL-cholesterol in CHD could be reformulated as: `what is the likely therapeutic benefit of targeting a specific protein (e.g. cholesteryl ester transfer protein, CETP) that influences HDL-cholesterol concentration?' Though the causal relevance of HDL-C in CHD is not directly answered by an analysis of this type because SNPs in the *CETP* gene also influence other major blood lipids and lipoproteins,^30^ these SNPs can help address the specific and important question of whether pharmacological modification of CETP to raise HDL-cholesterol will help prevent CHD events.^115^^,^^116^

## A guide to the selection of instruments for MR analysis of disease-associated biomarkers

Figure 3 summarizes some of the decisions to be made in the selection of instruments for MR analyses of disease biomarkers, based on the principles described in this review. These serve as a guide, but we emphasize that each MR analysis deserves thorough consideration on a case-by-case basis, with due attention paid to any underlying biological knowledge that may inform the design, analysis, reporting and inferences drawn.

For example, variants in the *IL6R* gene are associated with directionally opposite effects on CRP and interleukin-6, which may confuse interpretation of MR analysis using such instruments to evaluate the causal relevance of these two biomarkers. Insight comes from a comparison of the effect of pharmacological interleukin-6 receptor blockade on these biomarkers. This clearly shows that such variants mimic the effect of interleukin-6 receptor blockade and are variants optimally suited to a *cis*-MR of this receptor, with application in drug development.

The motivating factor for many MR analyses is the association between a biomarker and a disease outcome detected in an observational study. The next issue is whether any genetic variant(s) associated with the biomarker of interest have been identified that might serve as an instrument in an MR analysis. If the biomarker is a protein and SNPs can be identified in the encoding gene which influence its level or function, then a single locus *cis*-MR may be possible, provided confounding by LD and horizontal pleiotropy due to alternative splicing or miRNA effects can be confidently excluded, and the effect size is sufficiently large for an adequately powered analysis.

If the biomarker is not a protein and SNPs from multiple independent loci contribute to its variance, a multi-locus multi-instrument MR analysis may be possible, but the instrument is more likely to be affected by horizontal pleiotropy. The recent methodological advance of multivariable MR-Egger analysis may help deal with this. Alternatively, it may be possible to refocus the research question on variants influencing one or more of the proteins encoded by the loci influencing the biomarker of interest, that is reformulating the question as a *cis*-MR analysis.

Regardless of the approach used, consideration should be given to maximizing the sample size through the use of meta-analysis and the incorporation of public domain summary level estimates where possible.

## Conclusions

MR offers novel opportunities for reliable causal inference within the framework of observational research designs. The findings can provide insight into the pathophysiology of complex disease and have translational relevance, including the prioritization of drug targets. The emerging genetic architecture of disease biomarkers now allows more informed selection of genetic variants for MR studies than was hitherto possible. As the number of biomarker-associated variants grows, selection of the most appropriate instruments for MR analysis will become an increasingly important issue. We have proposed a set of principles that should inform the selection process to aid the design, analysis and interpretation of MR studies.

## Funding

D.I.S. is supported by a National Institute of Health Research Academic Clinical Fellowship. R.S. has been supported by a British Heart Foundation (Schillingford) Clinical Training Fellowship (FS/07/011). M.V.H. has been supported by a Medical Research Council Population Health Scientist Fellowship (G0802432). M.K. is supported by the National Institute on Aging (AG034454), the Medical Research Council (K013351), the National Heart, Lung and Blood Institute (HL036310) and the NordForsk. J.P.C. and A.D.H. are supported by University College London National Institute for Health Research Biomedical Research Centre. E.J.B. is supported by a British Heart Foundation programme grant (RG/13/2/30098) and the MooDFOOD Collaborative Project (FP7 grant 613598).

**Conflict of interest:** D I Swerdlow has been a consultant to Pfizer for work unrelated to this paper. John C Whittaker is employed by and holds stock in GSK.

## Supplementary Material

Supplementary Data
