# Supplementary material for: Selecting instruments for Mendelian randomization in the wake of genome-wide association studies
Source: Int J Epidemiol. 2016 Jun 24;45(5):1600–16. doi: 10.1093/ije/dyw088 (PMC5100611; doi:10.1093/ije/dyw088)

Supplementary Figure 1

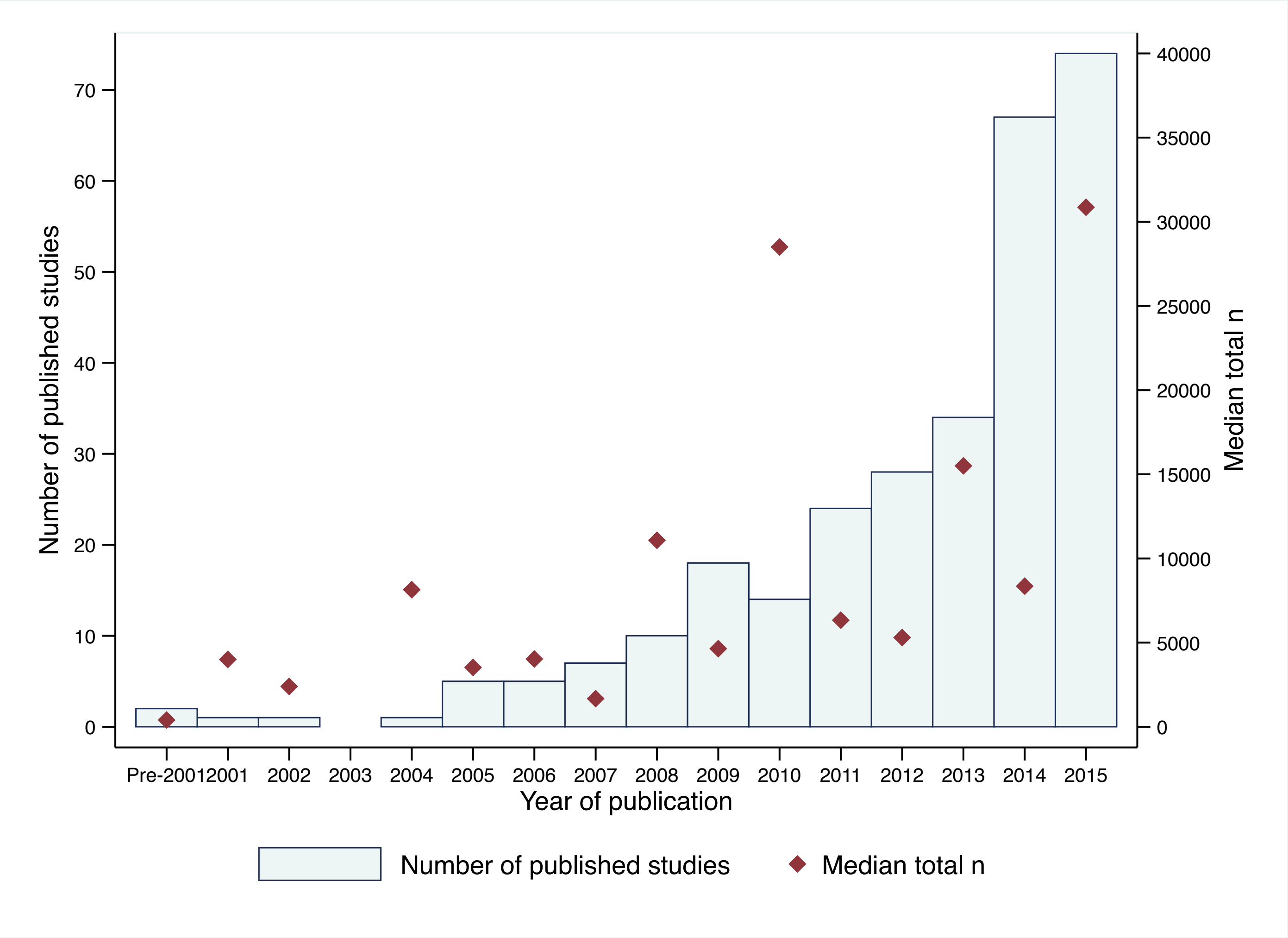

Supplementary Figure 2

a

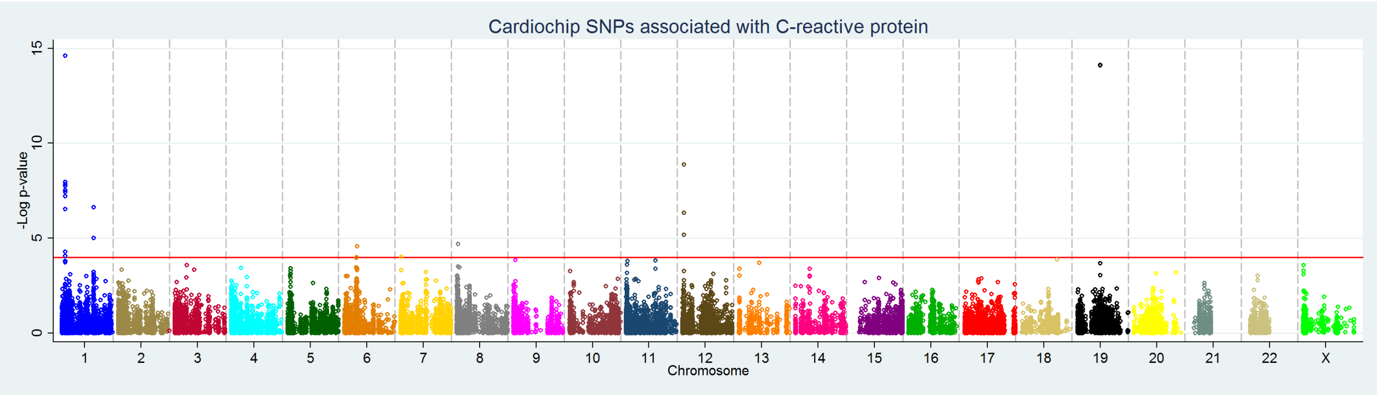

b

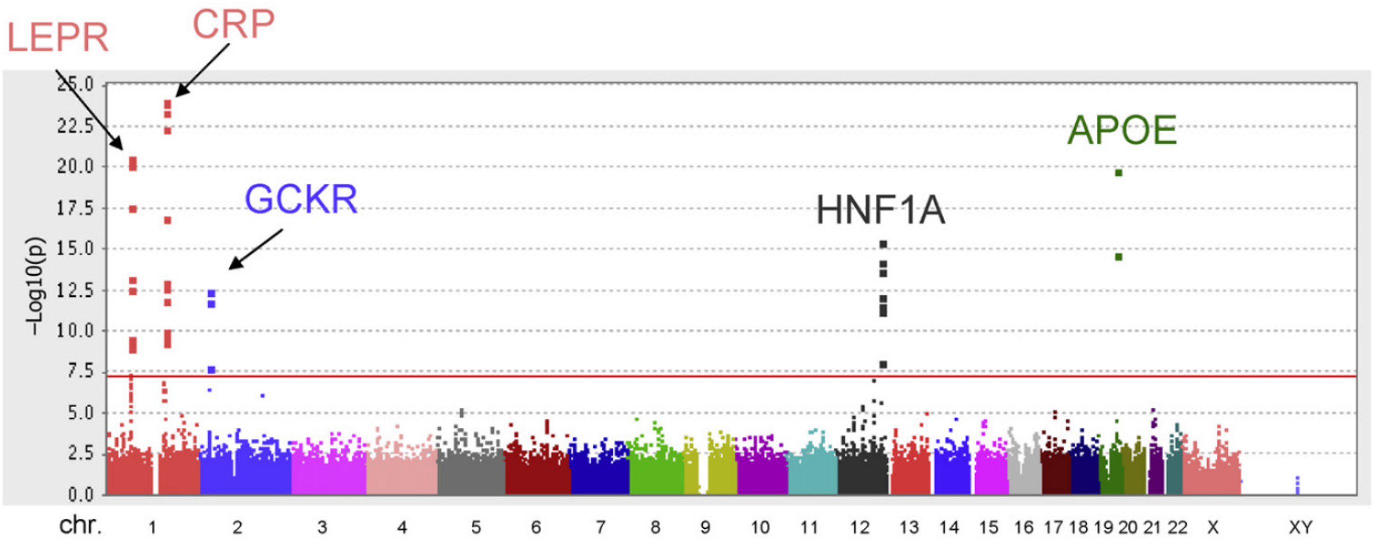

Ridker *et al. Am J Hum Genet* 2008

c

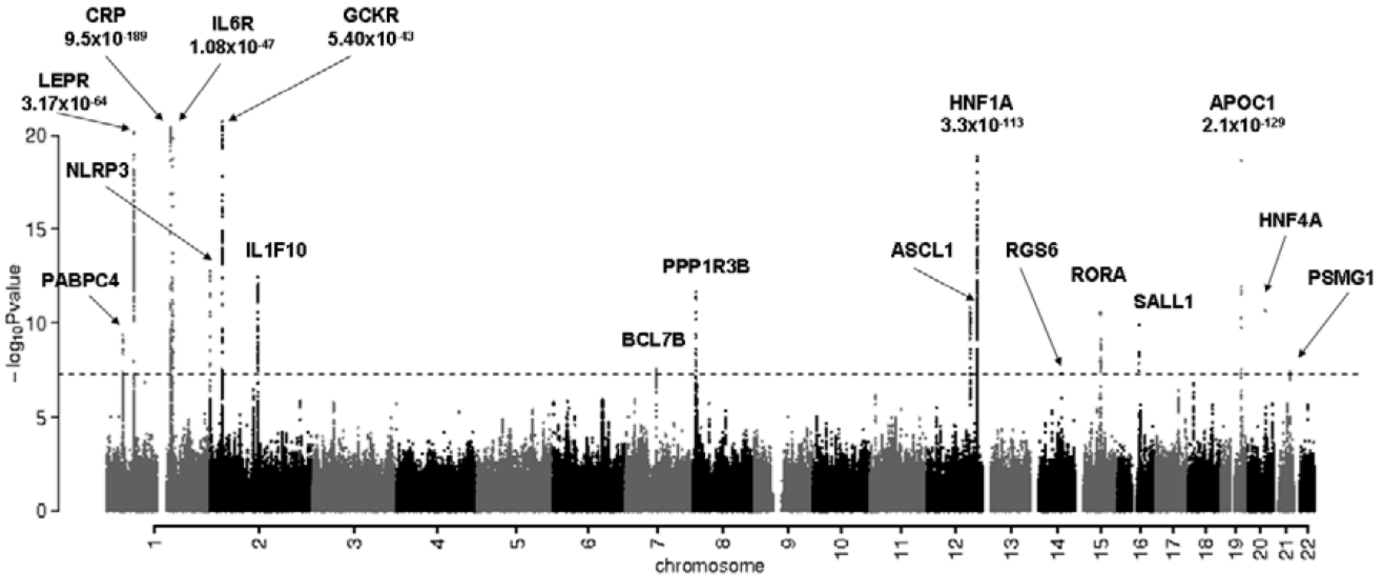

Dehghan *et al. Circulation* 2011

Supplementary Figure 3

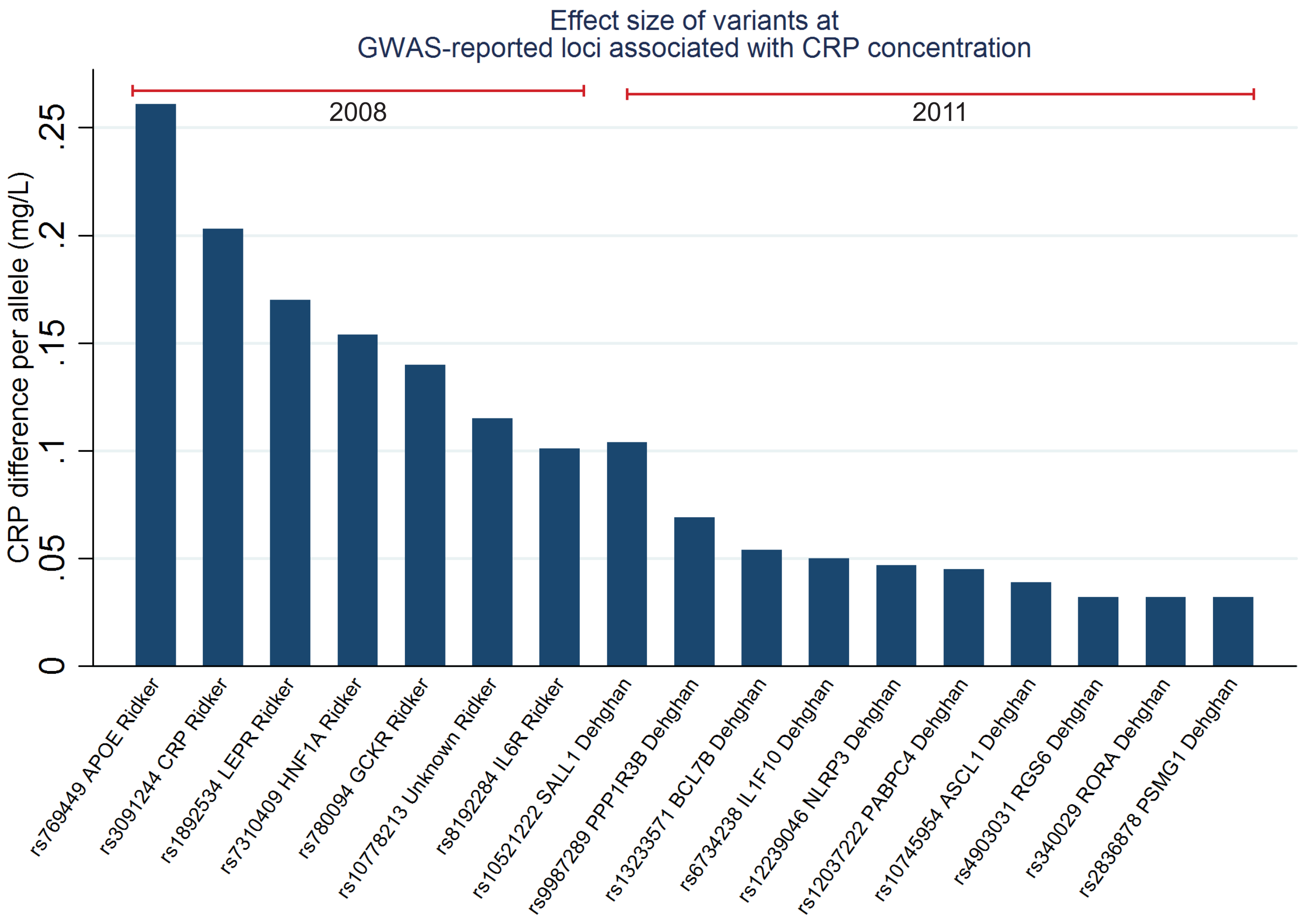

Supplementary Figure 4

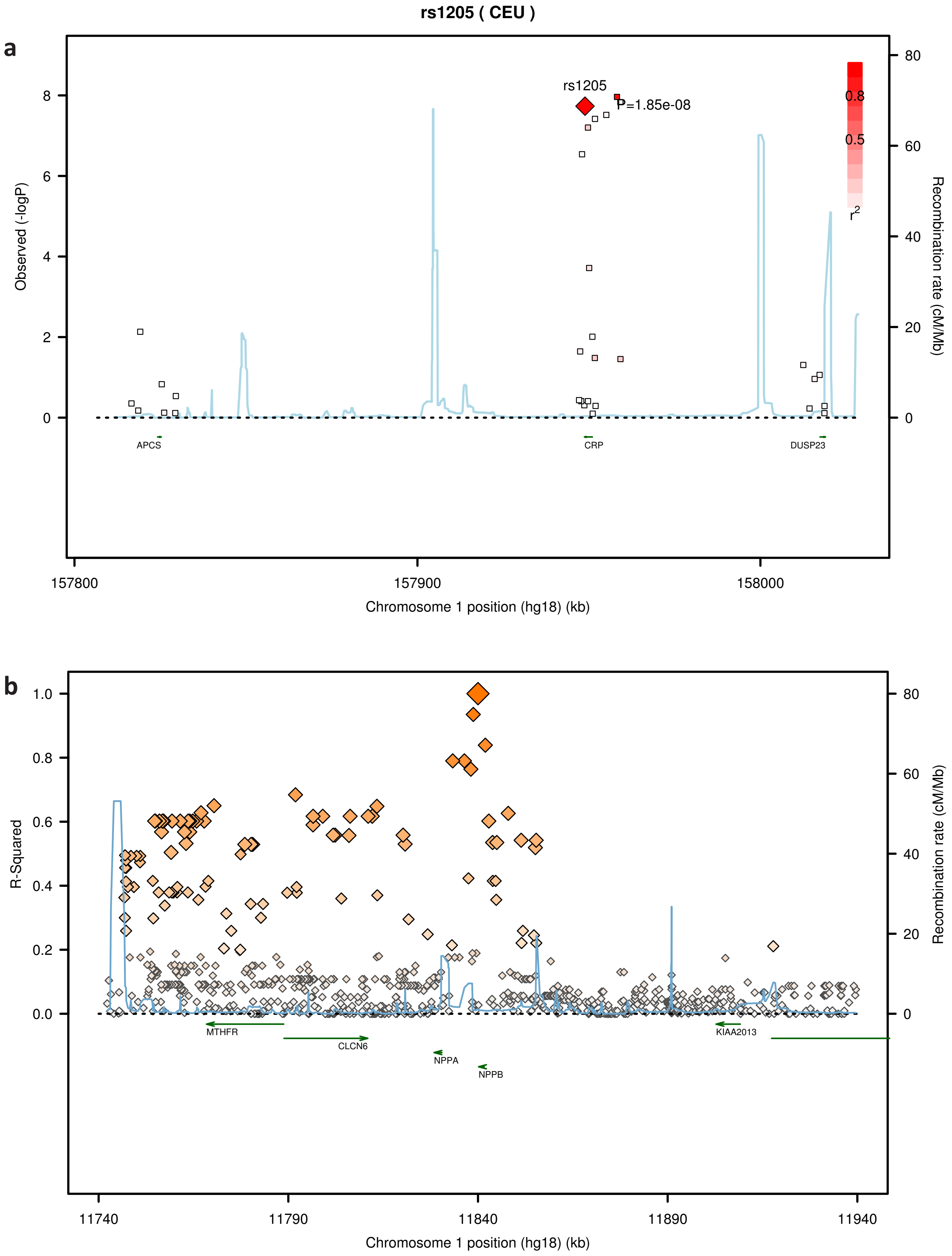

Supplementary Figure 5

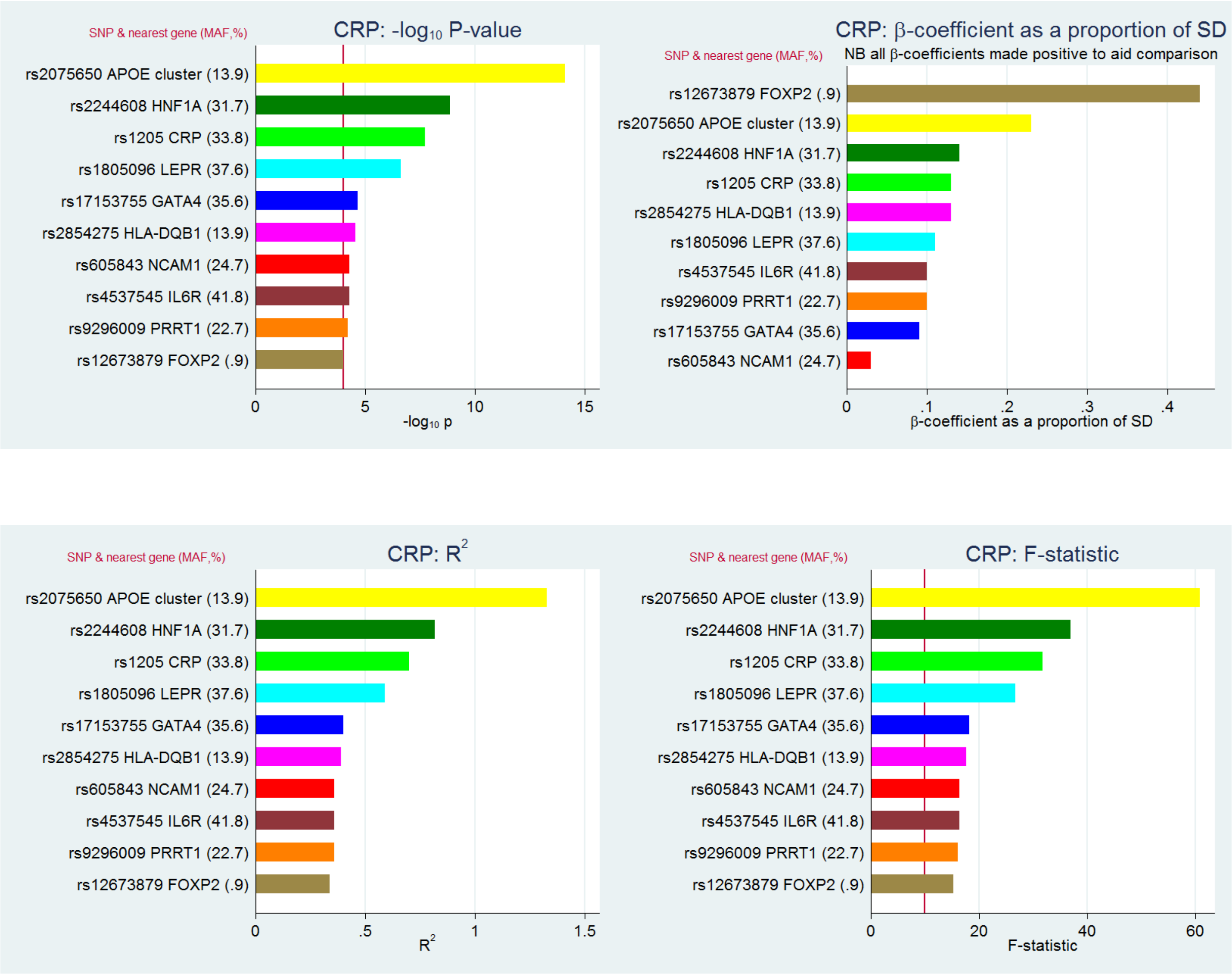

Supplementary Figure 6

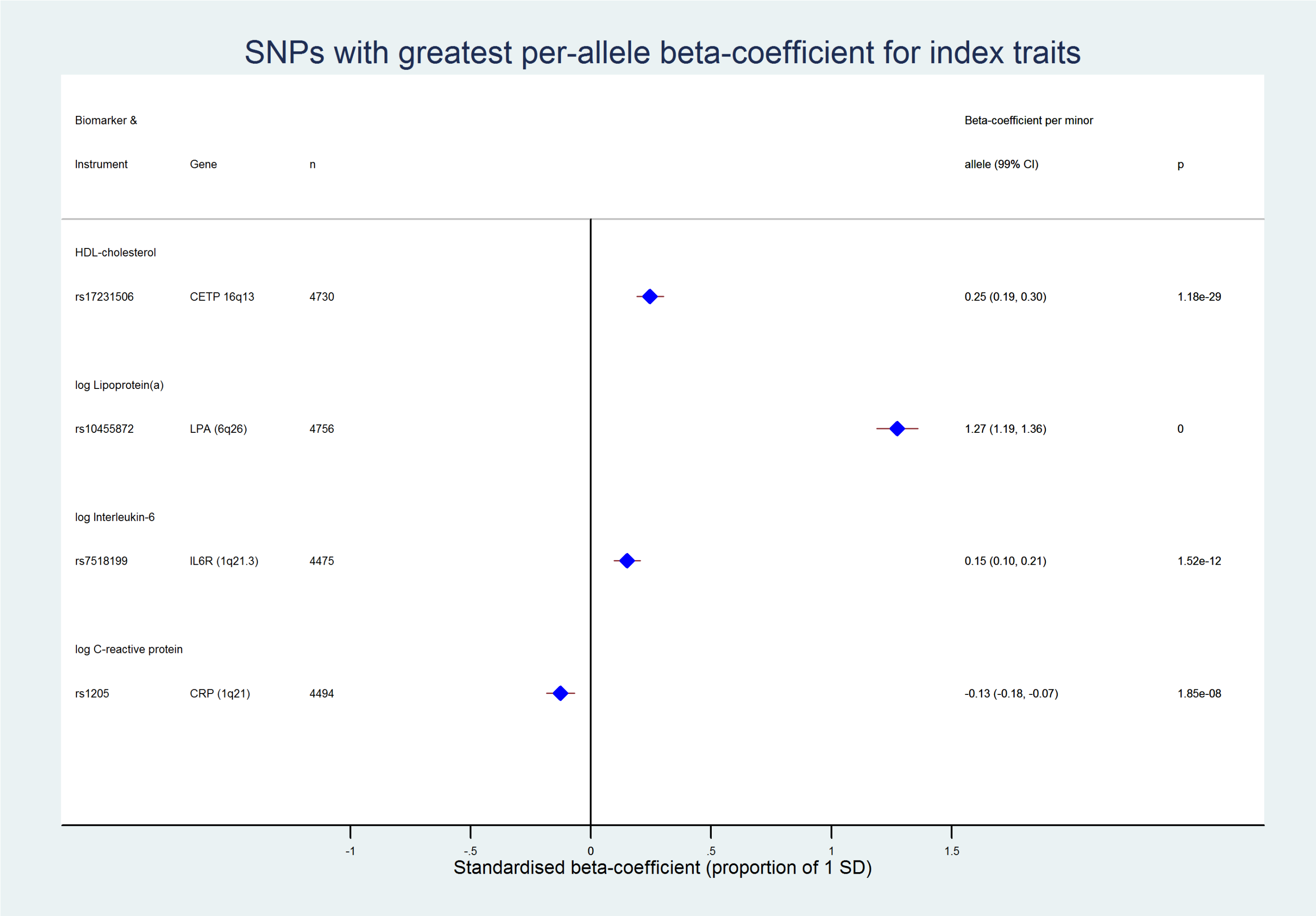

Supplementary Figure 7

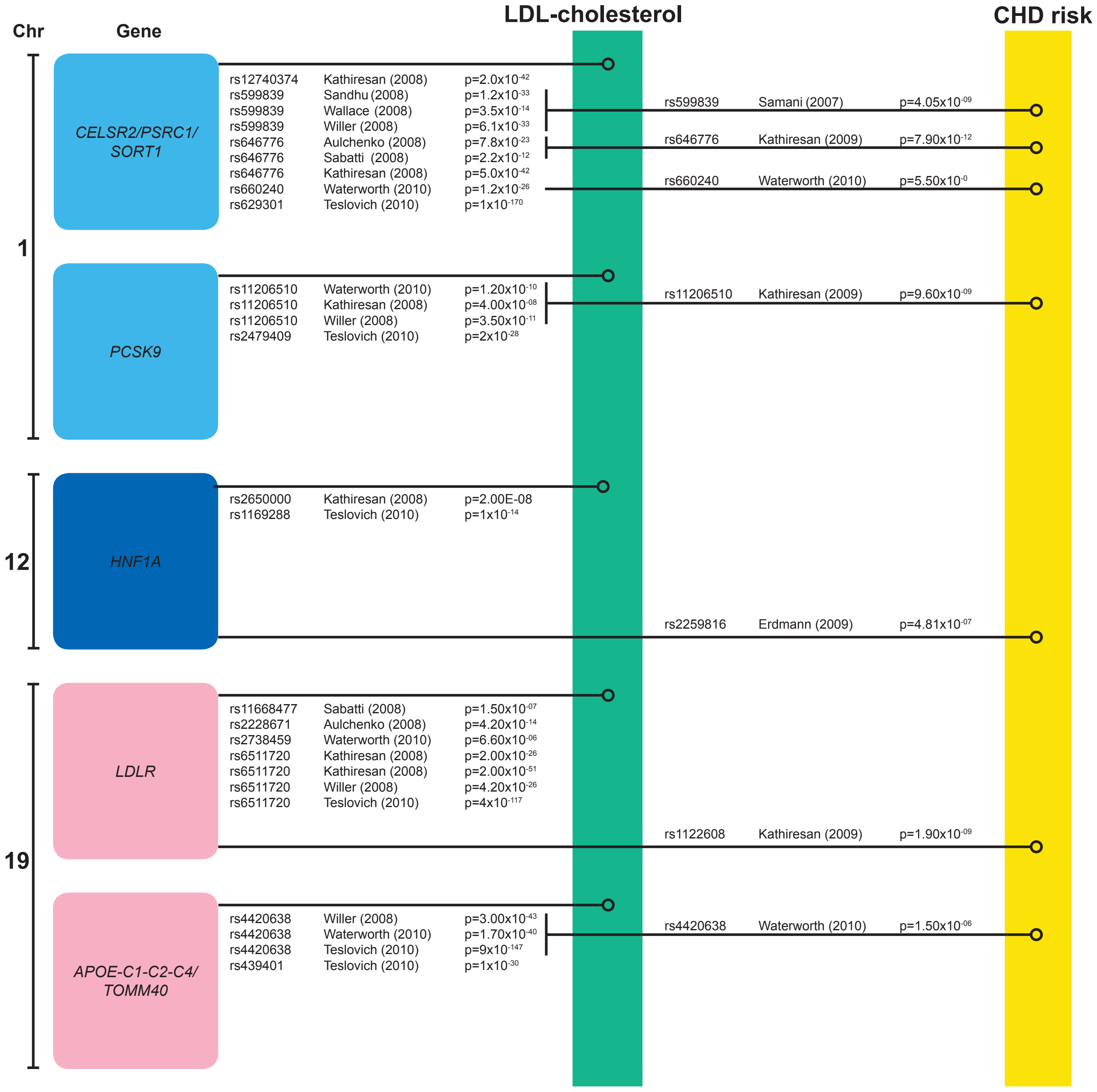

Supplementary Figure 8

a - Allele score for HDL-cholesterol in Whitehall II

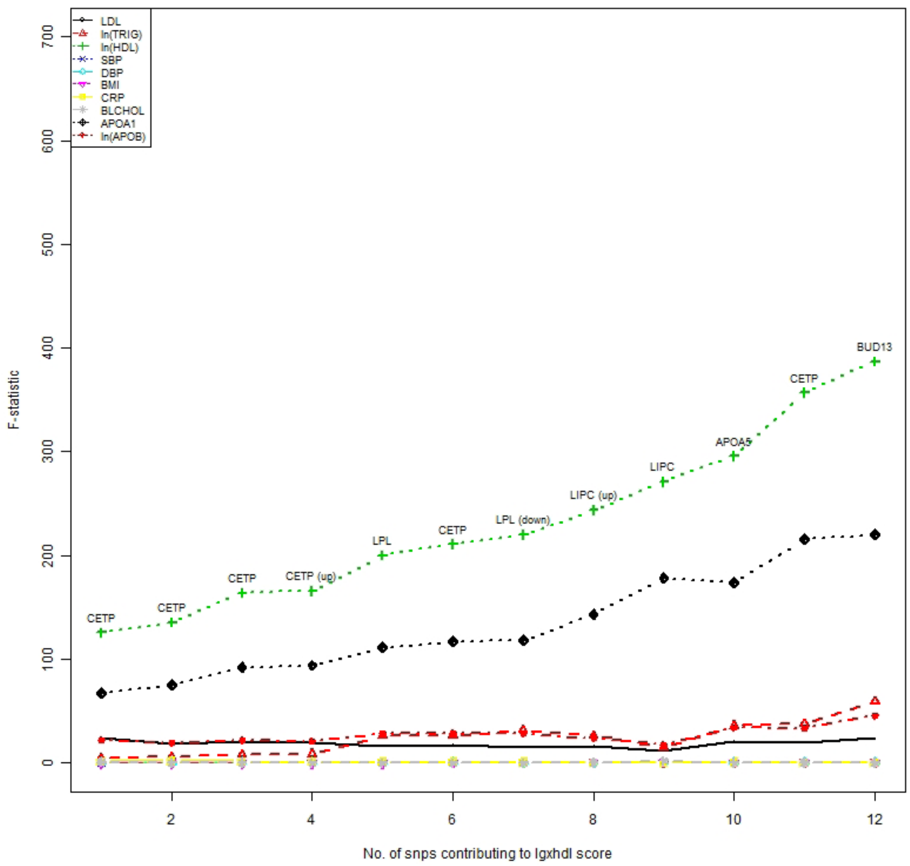

b - Allele score for triglycerides in Whitehall II

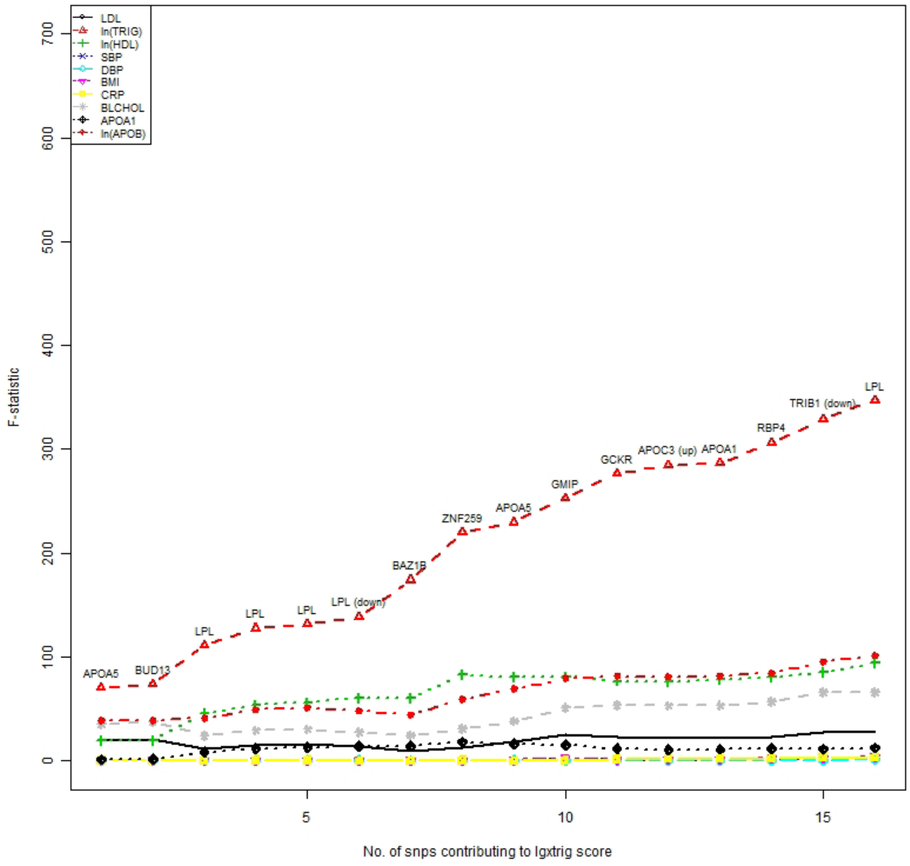

Supplement: Supplementary Data [file dyw088_supplementary_data.zip › ije-2014-10-1246-File006.pdf]
