# Supplementary material for: Selecting instruments for Mendelian randomization in the wake of genome-wide association studies
Source: Int J Epidemiol. 2016 Jun 24;45(5):1600–16. doi: 10.1093/ije/dyw088 (PMC5100611; doi:10.1093/ije/dyw088)
Supplement: Supplementary Data [file dyw088_supplementary_data.zip › ije-2014-10-1246-File005.docx]

#### Selecting genetic tools for Mendelian randomisation in the wake of genome-wide association studies

Swerdlow DI *et al.*

**Supplementary material**

**Supplementary methods**

**Literature search**

Primary studies and meta-analyses were included if they were published before 30 July 2015 and explicitly used genes to make inferences about the causal effect of an exposure on an outcome. The implementation of the instrumental variable (IV) approach was not a necessary criterion, however.

Medline was searched using the full text terms “Mendelian Randomization”, “Mendelian Randomisation”, and “Instrumental Variable” together with genetic*. The search retrieved 530 citations, which were reviewed, and a subset of 231 were deemed appropriate for inclusion.

**Gene-centric chip genetic association analysis of CRP**

*Whitehall II study – participant characteristics*

In brief, a cohort of 10,308 civil servants was recruited in 1985 from 20 UK government departments, with follow-up by regular questionnaire and clinical assessment including demographic, socio-economic, biometric, biochemical and other biological measures^1^. A DNA repository was established from follow-up phase 7 from 6,156 individuals and data on biomarkers and non-genetic exogenous exposures for the current analysis from phases 3 (1991-1994) and 7 (2002-2004). The study was approved by the UCL Research Ethics Committee, and participants gave informed consent to each aspect of the study.

*Biomarker measurement*

CRP was measured in serum stored at -80^o^C using a high-sensitivity immunonephelometric assay in a BN ProSpec nephelometer (Dade Behring, Marburg, Germany). Values of CRP below the detection limit (0.154mg/L) were assigned a nominal value of 0.077mg/L, half the lower detection limit. Interleukin-6 was measured using a high-sensitivity ELISA assay (R&D Systems, Oxford, UK), and values below the detection limit (0.08pg/ml) were assigned a value of 0.04pg/mL^2^. Fasting total and HDL-cholesterol were measured within 72 hours of venepuncture in serum stored at 4^o^C via standard enzymatic colorimetric methods in the same laboratories with identical methodology for all subjects. LDL-cholesterol concentration was calculated using the Friedewald formula. Levels of apolipoproteins A1 an B were determined b immunoturbidimetry. Systolic and diastolic blood pressure were measured twice in the sitting position after 5 minutes of rest with the Hawksley random-0 sphygmomanometer. To calculate body mass index (BMI), weight was measured in underwear to the nearest 0.1kg, and height to the nearest 1mm in bare feet using a stadiometer with the subject standing erect with the head in the Frankfort plane.

*Genotyping quality control*

Initial genotype calling with Illumina BeadStudio (Illumina, San Diego, USA) resulted in call frequencies below 98% for 1,798 of the 49,094 SNPs. These were reclustered manually, after which those remaining below 98% were discarded. Of 115 duplicate pairs, 113 had a concordance rate of over 99.5%, 1 of 99.1% and 1 of 95%, though manual reclustering resulted in 100% duplicate concordance. 5,557 samples passed the 80% pre-specified sample call rate. Of the 5,441 samples remaining after removal of duplicates and 1 case of ambiguous identity, 5,067 were individuals of white European ancestry. Principle component analysis in PLINK of genome-wide identity-by-descent identified 8 outliers who were also excluded, leaving 5,059 individuals. The final marker set included 48,032 SNPs with call rate above 98%. Genomic inflation factors for all the analyses reported were close to 1, indicating negligible influence from population structure or genotyping error.

**Supplementary figure legends**

**Supplementary Figure 1**

Total number of Mendelian randomisation studies published per year (2004-2014) and their median sample size (in 1000s).

**Supplementary Figure 2**

Genetic loci identified by genome-wide association studies for their association with CRP level (Manhattan plots)

a - Loci identified from analysis in the Whitehall II study using IBC HumanCVD BeadChip (n=5,059)

b - Loci identified by early GWA-study (n=6,345)^3^

c - Loci identified by later meta-analysis of GWA studies (n=82,725)^4^

**Supplementary Figure 3**

Effect size of CRP-associated variants identified by GWA studies declines in later, larger studies

**Supplementary Figure 4**

a - The *CRP* locus (chr1q23.2) showing SNPs associated with CRP levels in the Whitehall II study, p-values for the CRP association, linkage disequilibrium between SNPs (r^2^), and recombination rate.

b - SNPs at the *MTHFR-NPPA-NPPB* locus (chr1p36.2) showing linkage disequilibrium between SNPs (r^2^), and recombination rate.

**Supplementary Figure 5**

Ranking of CRP-associated SNPs in according to different measures of effect size (data from the Whitehall II study).

Ranking CRP-associated loci by p-value, proportion of trait variance explained (R^2^) and F-statistic results in similar ordering since all three metrics are statistically related and dependent on minor allele frequency. When ranked by standardised β-coefficient, the order of SNPs changes and may result in selection of a less appropriate variant for use in MR analysis.

**Supplementary Figure 6**

Wide variation in the effect size (standardised β-coefficient) of top-ranking SNPs associated with protein (CRP, IL-6) and non-protein (Lp(a), HDL-C) biomarkers. In the case of IL-6, SNPs in the *IL6* gene (chr7p15.3, i.e. *cis*-SNPs) did not show significant associations with circulating levels of the cytokine, which were best instrumented by variants in *IL6R* (chr1p21.3). For CRP levels, the most strongly associating SNPs were at the *APOE* cluster (chr19q13.32) but a *cis*-SNP in the *CRP* gene (chr1q23.2) was associated marginally less strongly and is included here.

**Supplementary Figure 7**

GWAS-identified SNPs and loci associated with LDL-cholesterol overlap with those associated with risk of coronary heart disease (CHD).

**Supplementary Figure 8**

The specificity and power of allele scores for non-protein biomarkers (a. HDL-C; b. triglycerides) increases as more variants are added to the score. Gene names are included above each data point. Where a gene name is repeated, this indicates the presence of additional SNPs exhibiting an independent association with the trait of interest at the same locus. For both HDL-C and triglyceride scores, the F-statistic for the association between score and index biomarker increases with addition of alleles while that for other lipid and non-lipid biomarkers remains low (with the exception of an expected rise for ApoA1 with the HDL-C score. The labelled gene symbols indicate each locus sequentially added to construct the score.

**Supplementary Table 1**

| **Organ/system** | **Altered biological variable (intermediate phenotype/risk factor/biomarker/quantitative trait)** | **Associated disease outcome** |
| --- | --- | --- |
|  |  |  |
| **Brain** | Cognitive function | Alzheimer’s disease, mental health disorders |
| **Heart** | Electrocardiogram (P-wave morphology, PR interval) | Atrial fibrillation |
|  | QT interval, QT duration, QT dispersion, heart rate variability | Sudden death |
| **Blood** | Circulating blood markers e.g. blood lipids and apolipoproteins, inflammation and coagulation markers | Atherosclerotic vascular disease (vascular dementia, stroke, coronary heart disease, peripheral vascular disease) |
| **Blood vessel** | Blood pressure (BP) carotid intima-media thickness (CIMT), flow mediated dilation (FMD), ankle-brachial pressure index (ABI). | Atherosclerotic vascular disease (vascular dementia, stroke, coronary heart disease, peripheral vascular disease) |
| **Lung** | FEV1, FVC, PEFR | Chronic obstructive pulmonary disease (COPD) |
| **Kidney** | Serum creatinine, eGFR, micro/macroalbuminuria | End stage renal disease (ESRD) |
| **Liver** | Blood markers: aspartate and alanine aminotranferase, gamma glutamyl transferase  Liver ultrasound: fatty change | Fatty liver disease and cirrhosis |
| **Bone** | Bone mineral density (BMD) | Osteoporotic fracture |
| **Endocrine** | Glucose, insulin, HbA_1_C, HOMA, BMI, WHR | Type 2 diabetes mellitus (T2DM), obesity |
| **Coagulation** | Coagulation factors | Deep vein thrombosis and pulmonary embolism |
|  |  |  |

**Phenotypic alterations that precede common diseases**

BMD=bone mineral density; BMI=body mass index; eGFR=estimated glomerular filtration rate; FEV=forced expiratory volume; FVC=forced vital capacity; HbA1c=glycosylated haemoglobin; HOMA=homeostasis model assessment; PEFR=peak expiratory flow rate; WHR=waist-hip ratio

**Supplementary Table 2**

| **Trait** | **F-statistic from regression of lipid trait on score** | **Largest F-statistic from regression on single SNP** | **Smallest F-statistic from regression on single SNP** |
| --- | --- | --- | --- |
| LDL-C score | 687 | 215 (*APOE* haplotype) | 14 (rs283813, *PVRL2*) |
| HDL-C score | 387 | 125 (rs17231506, *CETP)* | 20 (rs11820589, *BUD13*) |
| Triglycerides score | 347 | 71 (rs651821, *APOA5*) | 17 (rs3289, *LPL*) |

Increased association of multi-locus gene score with lipid traits compared to single SNPs

**Supplementary references**

1. Marmot M, Brunner E. Cohort Profile: the Whitehall II study. *Int J Epidemiol*. 2005 Apr;**34**(2):251–256.

2. Elovainio M, Ferrie JE, Singh-Manoux A, et al. Organisational justice and markers of inflammation: the Whitehall II study. *Occup Environ Med*. 2010 Feb;**67**(2):78–83.

3. Ridker PM, Pare G, Parker A, et al. Loci related to metabolic-syndrome pathways including LEPR,HNF1A, IL6R, and GCKR associate with plasma C-reactive protein: the Women’s Genome Health Study. *Am J Hum Genet*. 2008 May;**82**(5):1185–1192.

4. Dehghan A, Dupuis J, Barbalic M, et al. Meta-analysis of genome-wide association studies in >80 000 subjects identifies multiple Loci for C-reactive protein levels. *Circulation*. 2011 Feb 22;**123**(7):731–738.
